# Hyperglycemia induces PFKFB3 overexpression and promotes malignant phenotype of breast cancer through RAS/MAPK activation

**DOI:** 10.1186/s12957-023-02990-2

**Published:** 2023-03-28

**Authors:** Xiao Cheng, Xiupeng Jia, Chunnian Wang, Shangyan Zhou, Jiayi Chen, Lei Chen, Jinping Chen

**Affiliations:** 1 Department of Histopathology, Ningbo Clinical Pathology Diagnosis Center, Ningbo, 315000 Zhejiang China; 2 Department of Experimental Pathology, Ningbo Clinical Pathology Diagnosis Center, Ningbo, 315000 Zhejiang China; 3 Department of Cytopathology, Ningbo Clinical Pathology Diagnosis Center, Ningbo, 315000 Zhejiang China

**Keywords:** PFKFB3, miR-26, RAS/MAPK, Hyperglycemia, Breast cancer

## Abstract

**Background:**

Breast cancer is the most common tumor in women worldwide. Diabetes mellitus is a global chronic metabolic disease with increasing incidence. Diabetes mellitus has been reported to positively regulate the development of many tumors. However, the specific mechanism of hyperglycemic environment regulating breast cancer remains unclear. PFKFB3 (6-phosphofructose-2-kinase/fructose-2, 6-bisphosphatase 3) is a key regulatory factor of the glycolysis process in diabetes mellitus, as well as a promoter of breast cancer. So, we want to explore the potential link between PFKFB3 and the poor prognosis of breast cancer patients with hyperglycemia in this study.

**Methods:**

Cell culture was utilized to construct different-glucose breast cancer cell lines. Immunohistochemistry was adopted to analyze the protein level of PFKFB3 in benign breast tissues, invasive ductal carcinoma with diabetes and invasive ductal carcinoma without diabetes. The Kaplan–Meier plotter database and GEO database (GSE61304) was adopted to analyze the survival of breast cancer patients with different PFKFB3 expression. Western blot was adopted to analyze the protein level of PFKFB3, epithelial–mesenchymal transition (EMT)-related protein and extracellular regulated protein kinases (ERK) in breast cancer cells. Gene Set Cancer Analysis (GSCA) was utilized to investigate the potential downstream signaling pathways of PFKFB3. TargetScan and OncomiR were utilized to explore the potential mechanism of PFKFB3 overexpression by hyperglycemia. Transfections (including siRNAs and miRNA transfection premiers) was utilized to restrain or mimic the expression of the corresponding RNA. Cell functional assays (including cell counting, MTT, colony formation, wound-healing, and cell migration assays) were utilized to explore the proliferation and migration of breast cancer cells.

**Results:**

In this study, we demonstrated that the expression of PFKFB3 in breast cancer complicated with hyperglycemia was higher than that in breast cancer with euglycemia through cell experiment in vitro and histological experiment. PFKFB3 overexpression decreased the survival period of breast cancer patients and was correlated with a number of clinicopathological parameters of breast cancer complicated with diabetes. PFKFB3 promoted the proliferation and migration of breast cancer in a hyperglycemic environment and might be regulated by miR-26. In addition, PFKFB3 stimulated epithelial-mesenchymal transition of breast cancer in a hyperglycemic environment. In terms of downstream mechanism exploration, we predicted and verified the cancer-promoting effect of PFKFB3 in breast cancer complicated with hyperglycemia through RAS/MAPK pathway.

**Conclusions:**

In conclusion, PFKFB3 could be overexpressed by hyperglycemia and might be a potential therapeutic target for breast cancer complicated with diabetes.

## Introduction

Cancer is the second leading cause of death in human and often leads to panic worldwide [1]. According to the Global Cancer Observatory (https://gco.iarc.fr/), nearly 19.29 million people were diagnosed with cancer for the first time and nearly 10 million died from it in 2020. Among them, the number of breast cancer cases were 2.26 million (11.7%) and increased by 0.1% compared with the same period of 2018. Breast cancer-related deaths accounted to 685,000 in 2020, ranking fifth (6.9%) among all cancer-related deaths and increased by 0.3% compared with the same period of 2018 [2]. Breast cancer is also the most common cause of cancer-related deaths in women, accounting for 15.5% of female cancer-related deaths in 2020. As we all know, surgery combined with chemoradiotherapy is the primary treatment of breast cancer [3]. The emergence of hormone therapy and molecular targeted therapy in recent years is also an important milestone in the development of cancer therapy. However, proper targeting molecules remains urgently needed. Diabetes is a chronic metabolic disorder characterized by persistent hyperglycemia, among which type 2 diabetes accounts for about 95% and is the most common metabolic disease at present. According to the International Diabetes Federation (https://diabetesatlas.org/), the number of diabetics reached 536.6 million worldwide in 2021, accounting for 9.8% of the total population and 6.7 million of them died from diabetes. As the largest developing country, the proportion of diabetics in China reached 10.6% of the nation’s total population in 2021, higher than the global average level. In addition, the number of people with diabetes is predicted to reach 783.2 million in 2045, accounting for 11.2% of the population globally. Insulin resistance which prevents the body from using endogenous insulin effectively, has long been recognized as a cause of type 2 diabetes. A review published in Nature in 2019 by Gerald I. Shulman, a diabetes expert, suggested that the root cause of hyperglycemia is the increase of liver glycogen due to the abnormal white adipose tissue (WAT) degradation [4].

The relationship between cancer and diabetes, two major threats to human health, has been explored for decades. In 1924, German biochemist Otto Warburg first proposed the concept of Warburg effect, pointing out that tumor cells mainly meet the material needs of vigorous metabolism and rapid proliferation through glycolysis rather than tricarboxylic acid cycle, even though there is no lack of aerobic environment [5]. Statistical evidence indicated that hyperglycemia increased the incidence of multiple tumors and was associated with poor prognosis of cancer patients. 5-year overall survival was lower in tongue squamous cell carcinoma or stage IIIB-IV non-small cell lung cancer patients with diabetes than that in patients without diabetes [6, 7]. Studies have shown that hyperglycemic environment indirectly promoted the metastasis of tongue squamous cell carcinoma, pancreatic cancer, or gastric cancer by activating PKM2, HIF-1α, or ENO1, respectively [6, 8, 9]. A previous cancer prevention research based on one million Americans found that breast cancer patients with diabetes accounted for 16% of all cancer patients with diabetes [10]. Besides, according to the latest report, 18% of breast cancer patients also had diabetes [11]. Breast cancer patients with diabetes had a 24 to 44% higher risk of death than those without diabetes [12, 13]. Therefore, it is urgent to find appropriate targeting molecules for breast cancer patients with diabetes.

PFKFB3 (6-phosphofructose-2-kinase/fructose-2, 6-bisphosphatase 3), also known as PFK2, IPFK2 or iPFK-2, is a member of the fructose-2-kinase 6-phosphate/fructose-2, 6-diphosphatase family (PFKFB1-4) [14]. As a bifunctional protein, PFKFB3 can promote the synthesis and degradation of fructose-2, 6-bisphosphate (F2, 6BP, a key regulator of glycolysis) [15]. PFKFB3 is the most expressed PFKFB family gene in proliferating cells and cancer cells [16]. PFKFB3 is overexpressed in multiple solid tumors, including breast cancer, gastric cancer, colorectal cancer and pancreatic cancer [17–19]. Besides, the expression of PFKFB3 was reported to promote lymph node metastasis and increase the tumor node metastasis (TNM) stage [20]. The PFKFB3 promoter contains four response elements that bind to hypoxia-inducible factor (HIF-1α) [21], progesterone receptor (PR) [17], estrogen receptor (ER) [22] and serum response factor (SRF) [23] to facilitate gene transcription. In addition, insulin [19], inflammatory cytokines [24], transforming growth factor-β1 (TGF-β1) [25], lipopolysaccharide [26], and some other growth factors can also promote the expression of PFKFB3. The combination of PFKFB3 and PIM2 has been reported to increase the glucose level in breast cancer cells (glucose detection kit, Sigma, GAGO20) [27], so what role does PFKFB3 play in breast cancer patients with diabetes? What are the possible mechanisms?

In our study, PFKFB3 expression was firstly confirmed to be enhanced by mediums with high glucose concentration and the knockdown of PFKFB3 could inhibit the malignant phenotype of breast cancer. Then, the mechanisms of PFKFB3 upregulation by high glucose concentration and PFKFB3 promoting the malignant phenotype of breast cancer were explored by online databases. Cell experiment in vitro and histological experiment were also adopted to verify the results based on online databases. In general, we deduced that hyperglycemia might upregulate PFKFB3 expression by inhibiting miR-26 to promote the malignant phenotype of breast cancer.

## Methods

### Clinical samples

Paraffin-embedded sections of 40 cases of benign breast tissue, 80 cases of breast invasive ductal carcinoma with diabetes and 80 cases of breast invasive ductal carcinoma without diabetes were obtained from the Department of Histopathology of Ningbo Clinical Pathology Diagnosis Center (Ningbo, Zhejiang, China). The samples selected were all from the patients with breast tissue resection from 2016.01 to 2021.06. Breast cancer patients with no other underlying diseases that might affect the results of our study were included. All patients with diabetes had been diagnosed and fasting blood glucose was higher than 7.0 mmol/L at admission. Invasive lobular carcinoma and other less common types of breast cancer were excluded. The clinicopathological parameters were obtained from Electronic Medical Records and the pathological results. This study was reviewed by the ethics committee of Ningbo Clinical Pathology Diagnosis Center and was conducted in full accordance with the Declaration of Helsinki (Code of Ethics of the World Medical Association).

### Immunohistochemistry (IHC)

The expression level of PFKFB3 protein in the obtained 200 breast tissues was analyzed by immunohistochemistry using an UltraSensitive-SP kit (Maixin-Bio, Fuzhou, China). The operation was completely in accordance with the kit’s instructions. The specific schedule was as follows: primary antibody incubation time was 14–16 h (4 ℃); secondary antibody incubation time was 1 h (room temperature). Rabbit PFKFB3 polyclonal antibody (Cat No: 13763–1-AP) was purchased from Proteintech Group Inc. (Chicago, USA). The dilution concentration (1:200) recommended in the instructions for use has been verified by pre-test. The expression level of PFKFB3 was assessed by multiplying the staining intensity (0–3 points) and the percentage of nucleus-cytoplasmic staining cells (1: 0–25%, 2: 26–50%, 3: 51–75%, 4: 76–100%). A score of 0–6 was defined as low expression and a score of 7–12 as high expression [28]. The results of biopsy staining were synthesized after independent evaluation by chief physicians of the breast pathology subspecialty in the department of histopathology.

### Online databases

GEO (Gene Expression Omnibus, GSE61304, https://www.ncbi.nlm.nih.gov/geo/) and Kaplan–Meier plotter database were utilized to analyze the survival of breast cancer patients with different PFKFB3 expression. The data set GSE61304 contained the clinical parameters (including age, tumor grade, TNM stage, and survival period) and gene expression profile of 58 breast cancer patients, so the survival analysis based on it were relatively reliable and representative. TargetScan (https://www.targetscan.org/vert_80/), OncomiR (http://www.oncomir.org), and miRcode (http://www.mircode.org/index.php) were utilized to explore the potential mechanism of PFKFB3 overexpression by hyperglycemia. As an integrated platform for genomic, pharmacogenomic, and immunogenomic gene set cancer analysis, Gene Set Cancer Analysis (GSCA, http://bioinfo.life.hust.edu.cn/GSCA/#/expression) could be utilized to conduct the gene set enrichment analysis (GSEA) of multiple cancers. Moreover, the potential downstream signaling pathways of PFKFB3 could be investigated by GSEA to better reveal the mechanism.

### Cell culture

Human breast cancer cell lines BT474 and MCF-7 were purchased from ATCC (the American Type Culture Collection, Rockville, MD). The routine conditions for both cell cultures were RPMI 1640 glucose-free medium (Invitrogen, USA) containing 10% fetal bovine serum and 1% antibiotics (penicillin and streptomycin, FBS, Invitrogen, USA), cell incubator containing 5% CO2 at 37 ℃. Special treatments were: the mediums with 5.5 mM, 15 mM, or 25 mM glucose were confected with sterile glucose solution to culture the two breast cancer cell lines continuously until the cells were passed for 8 times, so as to simulate different blood glucose environments in human body and construct different-glucose breast cancer cell lines (cells cultured with different glucose concentrations were hereinafter referred to as BT474/MCF-7–5.5/15/25 mM (mmol/L)).

### Western blot (WB)

Protein levels of PFKFB3, E-cadherin, N-cadherin, Vimentin, total/phosphorylated-ERK 1/2 (t/p-ERK1/2), and β-actin were tested by western blot as narrated before [29]. The specific schedule was as follows: transmembrane (90 min, 300 milliampere); blocking with milk (45 min); primary antibody incubation time was 2 h; secondary antibody (1:10,000) incubation time was 1 h. Mouse monoclonal antibody against β-actin (1:50,000, Cat No.: 66009–1-Ig), E-cadherin (1:4000, Cat No.: 60335–1-Ig), N-cadherin (1:4000, Cat No.: 66219–1-Ig), Vimentin (1:50,000, Cat No.: 60330–1-Ig) and rabbit polyclonal antibodies against PFKFB3 (1:1000, Cat No.: 13763–1-AP), total-ERK 1/2 (t-ERK1/2, 1:1000, Cat No.: 11257–1-AP), and phosphorylated-ERK 1/2 (p-ERK1/2, 1:3000, Cat No.: 28733–1-AP) (all from Proteintech Group, USA) were adopted.

### Transfection

As previously narrated in our laboratory, the specific operations were as recommended by the guidelines [29]. Lip3000 (Invitrogen, USA) were selected as transfection reagents as recommended. All small interfering RNAs (siRNAs), including siPFKFB3-1, siPFKFB3-2, and siNC (negative control), and miRNA transfection primers (miR-26-mimics-NC, miR-26-mimic, miR-26-inhibitor-NC, and miR-26-inhibitor) were all designed and supplied by GenePharma (Shanghai, China). Specific siRNA or miRNA were siPFKFB3-1, 5′-GGAGACACAUGAUCCUUCATT-3′; siPFKFB3-2, 5′-GCAUCGUGUACUAC CUGAUTT-3′; siNC, 5′-UUCUCCGAACGUGUCACGUTT-3′; miR-26-mimic-NC, 5′-CAGUACUUUUGUGUAGUACAA-3′; miR-26-mimic: 5′-UUCAAGUAAUCC AGGAUAGGCU-3′; miR-26-inhibitor-NC, 5′-CAGUACUUUUGUGUAGUACAA -3′; miR-26-inhibitor, 5′-AGCCUAUCCUGGAUUACUUGA A-3′.

### Cell functional assays

The cell counting assay, MTT assay and colony formation assay (all as narrated earlier [30]) were used to assess the cellular proliferation. The wound-healing assay and cell migration assay (as narrated earlier [30]) was used to assess the cellular metastasis. In the cell counting assay, 1 × 10^5^ cells were taken as the initial value and planted into 6-well plate, the number of cells were counted and recorded at the same time for five consecutive days. In the MTT assay, 2000 cells (MCF-7) or 5000 cells (BT474) were taken as the initial value and planted into 96-well plate, the absorbance of cells at 570 nm (OD570nm) was monitored 72 h later. In the cell colony formation assay, 1500 MCF-7 cells or 2500 BT474 cells were taken as the initial value and planted into 6-well plate. Culture plates were collected and colony images were taken to count the number of cell colony containing more than 100 cells after 2 weeks. In the wound-healing assay, a 20-μl pipette suction was used to draw a straight line when the cells in the 6-well plate grew to 90% density and then the shed cells were washed off with PBS. The micrographs of wounds were taken with an Olympus microscope (Olympus, Tokyo, Japan) at 0 h and 24 h respectively to compare cell migration in different groups. In the migration assay, 1 × 10^5^ cells were taken as the initial number and mixed with medium (lack of FBS). Then the cellular mixture was added into the upper chambers, medium with 5% FBS was added into the lower chambers meanwhile. Images of cells in the upper chambers were collected 24 h after dyed with 0.1% crystal violet (A100528; Sangon Biotech) for 10 min.

### Statistical analysis

All experiments were repeated for more than three times and the average was finally showed in this study. The survival analyses were conducted with Kaplan–Meier curve. The correlation analysis of PFKFB3 expression and clinical parameters of breast cancer patients were performed with Pearson’s chi-squared test in SPSS 26.0. Unpaired two-tailed *t* tests was utilized to process the experimental results. *P* < 0.05 was considered statistically significant.

## Results

### PFKFB3 was correlated with the prognosis of breast cancer (BC) patients and its expression could be enhanced by hyperglycemia

**Fig. 1:**
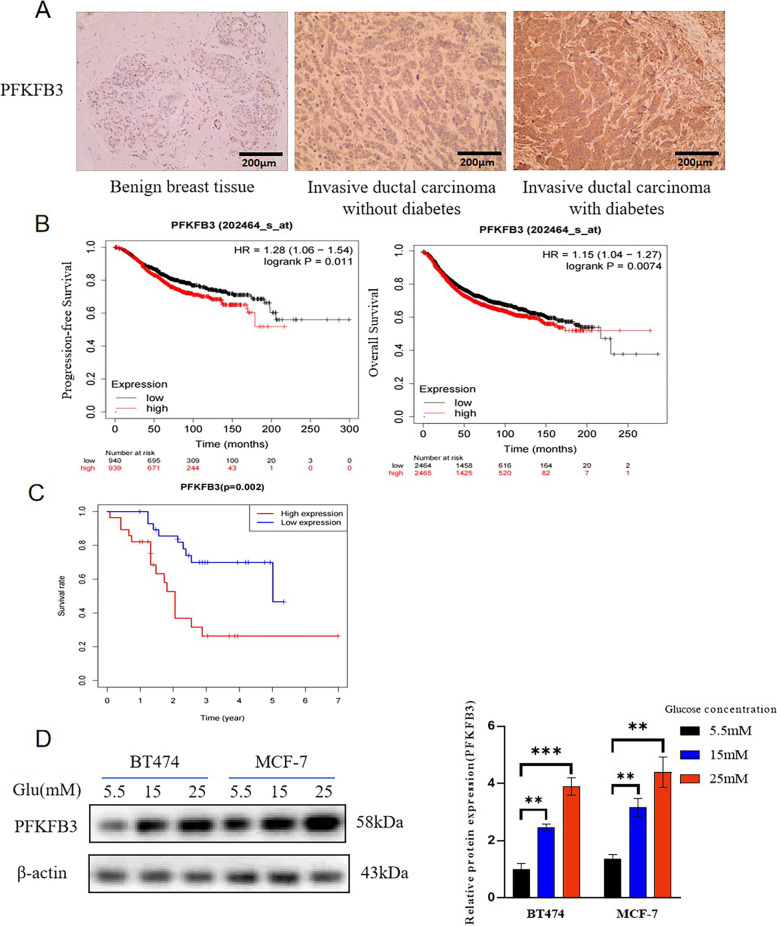
PFKFB3 could be enhanced by hyperglycemia and was correlated with the prognosis of BC patients. **A** Protein levels of PFKFB3 in benign breast tissues and invasive ductal carcinoma with or without diabetes were detected by IHC. The magnification of the photographs was 200. **B** The Kaplan–Meier plotter database and **C** GEO database (GSE61304) were utilized to compare the PFS and OS of breast cancer patients with different PFKFB3 expression levels. **D** The breast cancer cell lines (BT474 and MCF-7) were cultured in 1640 mediums with different concentrations of glucose (5.5 mM, 15 mM, or 25 mM) and WB was performed to evaluate PFKFB3 expression level. *, *P* < 0.05; **, *P* < 0.01; ***, *P* < 0.001

The PFKFB3 expression level of benign breast tissues and invasive ductal carcinoma with/without diabetes were compared with IHC based on the multi-center cases we collected to ensure the reliability of the results. The representative images of PFKFB3 in benign breast tissues and breast invasive ductal carcinoma with/without diabetes were shown in Fig. 1A. The results showed that the expression level of PFKFB3 in non-diabetic invasive ductal carcinoma was higher than that in benign breast tissue (*P* < 0.001), while lower than that in diabetic invasive ductal carcinoma patients (*P* = 0.031) (Table 1). PFKFB3 expression was significantly upregulated in breast cancer tissues. Likewise, the association of PFKFB3 expression with clinicopathological parameters from breast cancer patients were analyzed with correlation analysis and the results showed that PFKFB3 expression was relevant to blood glucose level (*P* < 0.001), lymph node metastasis (*P* = 0.005), and tumor stage (*P* < 0.001) (Table 2). The Kaplan–Meier plotter database was utilized to study the effect of PFKFB3 expression level on the survival of breast cancer patients. According to Fig. 1B, high PFKFB3 expression was correlated with poor progression-free survival (PFS, HR = 1.28, *P* = 0.011) and overall survival (OS, HR = 1.15, *P* = 0.0074). The survival analysis was also conducted with GEO database (GSE61304) to verify the results based on Kaplan–Meier plotter database and consistent results were obtained (Fig. 1C, *P* = 0.002). The results in Fig. 1D showed that PFKFB3 expression level was evidently enhanced with the glucose concentration of mediums increased while the expression of housekeeping protein (β-actin) was similar, suggesting that high glucose environment promoted the expression of PFKFB3 in breast cancer.

**Table 1:** Expression of PFKFB3 in benign breast tissues and invasive ductal carcinoma (with and without diabetes)

		PFKFB3 expression		*P*	*χ*^2^
Group	*n*	Low, *n* (%)	High, *n* (%)		
Benign breast tissues	40	31 (77.5)	9 (22.5)		
Invasive ductal carcinoma without diabetes	80	46 (57.5)	34 (42.5)	< 0.001^a^	14.731
Invasive ductal carcinoma with diabetes	80	22 (27.5)	58 (72.5)	0.031^b^	4.639

^a^Benign breast tissues and Invasive ductal carcinoma without diabetes

^b^Invasive ductal carcinoma with and without diabetes

**Table 2:** Association of PFKFB3 expression with clinicopathological parameters from breast cancer patients

		PFKFB3 expression			
		(*n* (%))		*P*	*χ*^2^
Parameter	*n*	Low	High		
Age (years)				0.618	0.249
≤ 50	83	41	42		
> 50	77	35	42		
Blood glucose (mmol/L)				< 0.001	25.664
≤ 7.0	80	54	26		
> 7.0	80	22	58		
Tumor size (cm)				0.501	0.452
≤ 2	63	32	31		
> 2	97	44	53		
Lymph node metastasis				0.005	8.035
No	78	46	32		
Yes	82	30	52		
Grade				0.954	0.003
I–II	126	60	66		
III	34	16	18		
Stage				< 0.001	38.202
I–II	92	63	29		
III–IV	68	13	55		

*P* < 0.05 was considered statistically significant

### PFKFB3 overexpression might activate epithelial–mesenchymal transition and RAS/MAPK pathways of breast cancer in a hyperglycemic environment

**Fig. 2:**
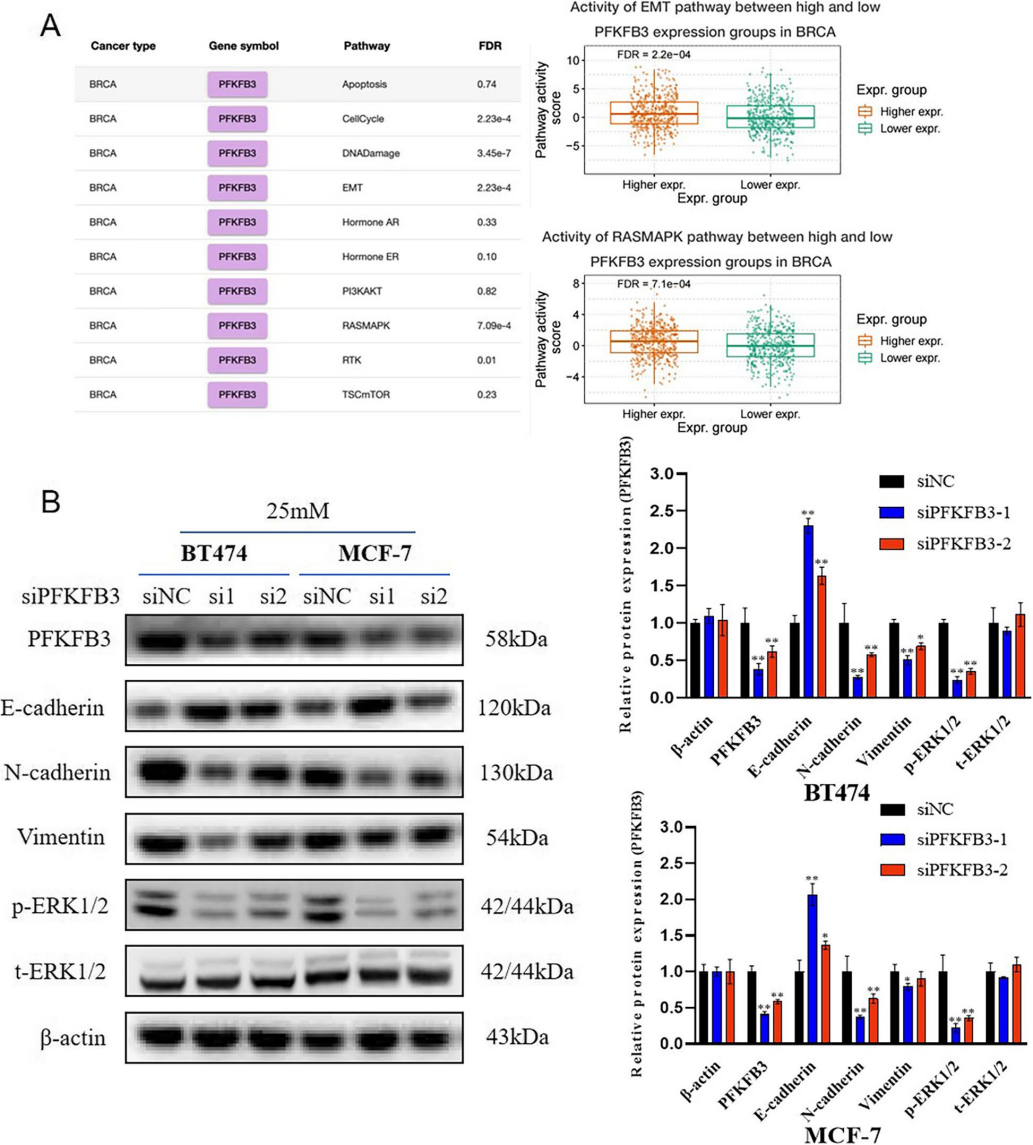
PFKFB3 might activate epithelial–mesenchymal transition and RAS/MAPK pathways of breast cancer in a hyperglycemic environment. **A** GSEA (Gene Set Enrichment Analysis) was performed to investigate the downstream signaling pathways. Pathway lists of PFKFB3 screened out by GSEA were shown on the left, the box plots of EMT (upper) and RAS/MAPK pathway (lower) were shown on the right. **B** Protein levels of PFKFB3, E-cadherin, N-cadherin, Vimentin, p-ERK1/2, and t-ERK1/2 in BT474-25 mM or MCF-7-25 mM cells after transfected with siPFKFB3-1, siPFKFB3-2 or siNC were detected using western blot. β-actin was detected as control. *, *P* < 0.05; **, *P* < 0.01

Given the crucial role of PFKFB3 in breast cancer progression, GSEA (Gene Set Enrichment Analysis) was performed to further investigate the downstream signaling pathways. According to Fig. 2A, RAS/MAPK (*P* < 0.001) and EMT (*P* < 0.001) were the most significant common pathways of enrichment in breast cancer. Briefly, the results indicated that PFKFB3 expression level might be correlated with the activation of RAS/MAPK and EMT pathways in breast cancer. According to Fig. 2B, PFKFB3 knockdown decreased the expression of N-cadherin, Vimentin and p-ERK1/2, while increased the expression of E-cadherin when the fluctuation of t-ERK1/2 and β-actin was not obvious. In other words, PFKFB3 knockdown inhibited the EMT and RAS/MAPK pathway of breast cancer in a hyperglycemic environment.

### PFKFB3 downregulation restrained the proliferation and migration of breast cancer in a hyperglycemic environment

**Fig. 3:**
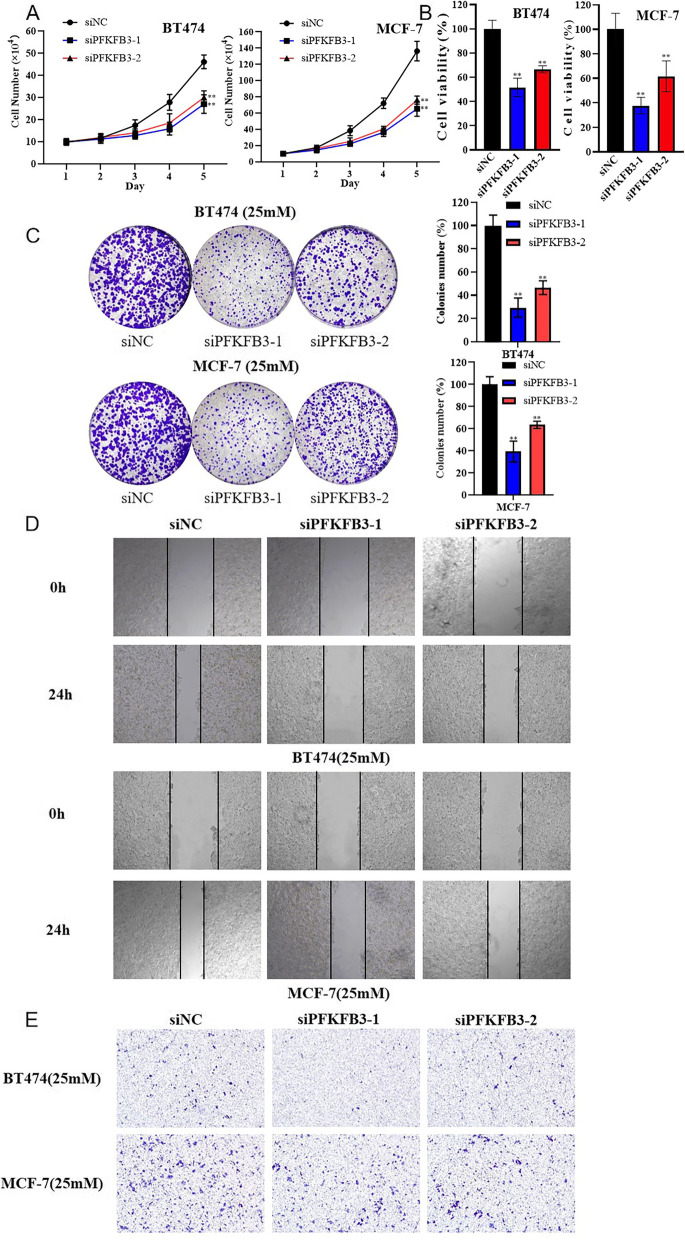
PFKFB3 promoted proliferation and migration of breast cancer cells in a hyperglycemic environment. BT474-25 mM and MCF-7-25 mM cells were transfected with siPFKFB3-1, siPFKFB3-2, or siNC. **A** The cell counting assay. **B** MTT assay. **C** The cell colony formation assay. **D** The wound-healing assay. **E** The migration assay

Besides, to investigate the role of PFKFB3 in the biological behaviors of breast cancer such as proliferation and migration in hyperglycemic environment, cell functional experiments were carried out. The results of cellular proliferation experiment (Fig. 3A: cell counting assay; Fig. 3B: MTT assay; Fig. 3C: colony formation assay) showed that the mitotic ability, cell viability and the capacity to form colonies were significantly decreased after PFKFB3 knockdown in breast cancer cells; the results of cellular migration experiment (Fig. 3D: wound-healing assay and Fig. 3E: migration assay) showed that the migration ability of breast cancer cells was significantly decreased after PFKFB3 knockdown. Briefly, PFKFB3 knockdown could remarkably suppress the proliferation and migration of breast cancer in a hyperglycemic environment.

### Hyperglycemia might promote PFKFB3 expression by miR-26 downregulation in breast cancer

**Fig. 4:**
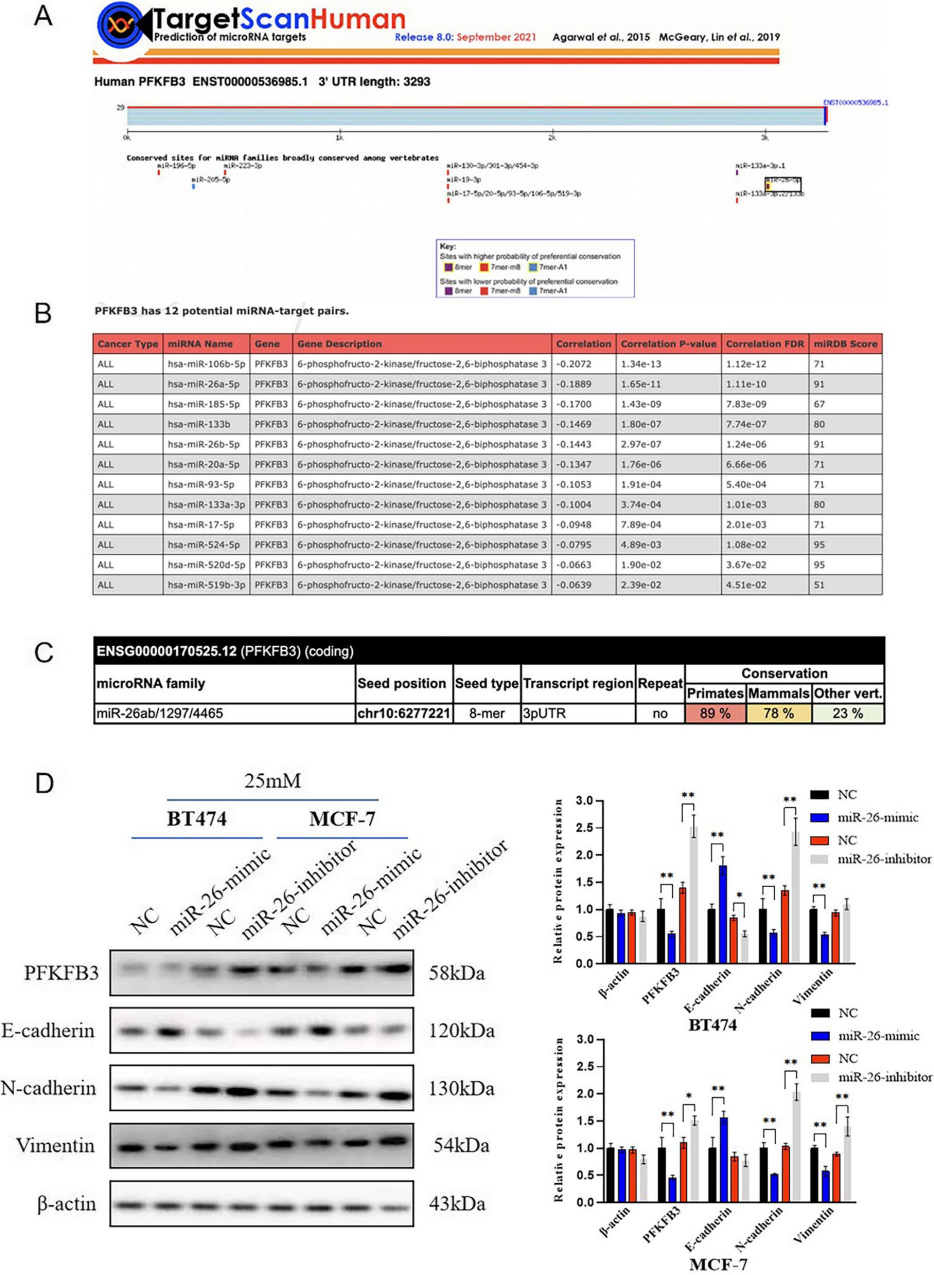
Hyperglycemia might promote PFKFB3 expression by miR-26 downregulation in breast cancer. **A** TargetScan database, **B** OncomiR database, and **C** miRcode database were utilized to predict that miR-26 was a reliable upstream microRNA of PFKFB3. **D** Protein levels of PFKFB3, E-cadherin, N-cadherin, and Vimentin in BT474-25 mM or MCF-7-25 mM cells after transfected with miR-26-mimic, miR-26-inhibitor, or corresponding-NC were detected by western blot. β-actin was detected as control. *, *P* < 0.05; **, *P* < 0.01

TargetScan database was utilized to explore the potential mechanism of PFKFB3 overexpression by hyperglycemia. According to Fig. 4A, miR-26 was the most conserved microRNA that regulates PFKFB3. In other words, miR-26 was the most possible upstream regulatory factor of PFKFB3. In OncomiR database, we also found that the correlation between PFKFB3 and miR-26 is relatively high, ranking only second to miR-106 (Fig. 4B). In miRcode database, the consistent result was obtained that PFKFB3 and miR-26 have a high degree of combination conservatism in primates or mammals (Fig. 4C). According to Fig. 4D, PFKFB3 expression was significantly enhanced in breast cancer cells transfected with miR-26-inhibitor, while miR-26-mimic decreased PFKFB3 expression. Besides, we also found that miR-26-inhibitor could promote the epithelial–mesenchymal transition of breast cancer cells, while miR-26-mimic had the opposite effect. Based on the results above, we could deduce that PFKFB3 overexpression by hyperglycemia might be by the way of miR-26 downregulation in breast cancer.

### miR-26 downregulation accelerated the proliferation and migration of breast cancer in a hyperglycemic environment

**Fig. 5:**
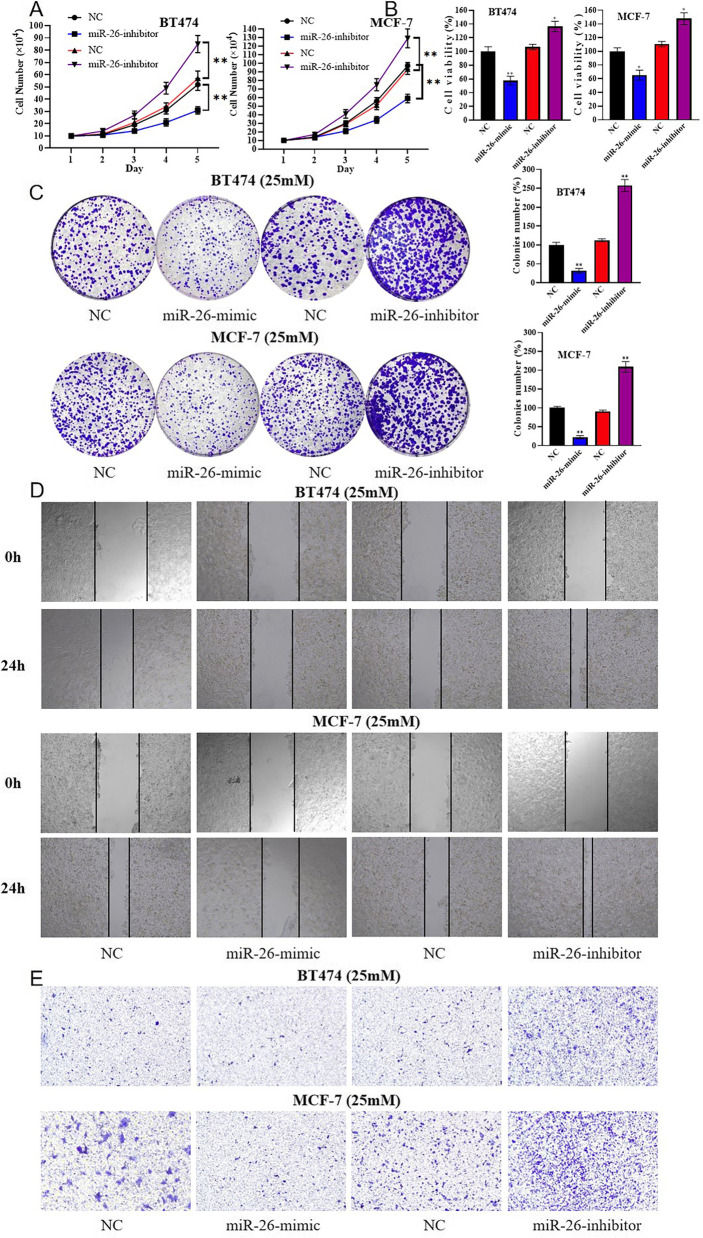
miR-26 downregulation accelerated the proliferation and migration of breast cancer in a hyperglycemic environment. BT474-25 mM and MCF-7-25 mM cells were transfected with miR-26-mimic, miR-26-inhibitor, or corresponding-NC. **A** The cell counting assay. **B** MTT assay. **C** The cell colony formation assay. **D** The wound-healing assay. **E** The migration assay. **, *P* < 0.01

Cell functional experiment was conducted to better reveal the effect of miR-26 on breast cancer. In cell counting assay (Fig. 5A), MTT assay (Fig. 5B) and colony formation assay (Fig. 5C), the proliferation was significantly promoted by miR-26-inhibitor, while miR-26-mimic had the opposite effect. In wound-healing assay (Fig. 5D) and migration assay (Fig. 5E), miR-26-inhibitor enhanced the migration of BT474 and MCF-7 cultured in medium with 25 mM glucose, while miR-26-mimic had the opposite effect. Briefly, miR-26 inhibited the proliferation and migration of breast cancer cells in a hyperglycemic environment.

## Discussion

With the alteration of human living standard and lifestyle, metabolic syndrome represented by hyperglycemia has gradually become a serious global health concern. According to statistics, about 10% of the world’s population is diabetic. Besides, as a well-known global chronic killer, one person dies of diabetes every 5 s and the damage seems to be more severe in developing countries [31]. Could there be a link between this invisible damage and the more familiar visible damage of cancer? The hyperglycemic environment has been proved to promote the occurrence and development of gastric cancer, colorectal cancer, hepatocellular carcinoma, pancreatic cancer, and lung cancer through a variety of signaling pathways [8]. As the most common tumor in women, breast cancer have also been found to be adversely affected by hyperglycemia in occurrence and progression [13, 32, 33]. Metformin is the first-line drug for the treatment of diabetes that can effectively decrease the blood glucose level [34], what is more exciting is that it can enhance the therapeutic effect of cancer treatment [35]. This discovery plays a positive role in improving the prognosis of breast cancer patients with hyperglycemia, which also provides new sights for cancer treatment: inhibiting tumor biological behaviors by blocking or attenuating glycolysis activity.

As we all know, the rate-limiting step in glycolysis determines the metabolic efficiency of carbohydrates. Fructose-6-phosphate is converted to fructose-1, 6-bisphosphate under the unidirectional catalysis of 6-phosphofructokinase-1 (PFK-1), which is irreversible and therefore an essential rate-limiting step in glycolysis. PFK-1 is therefore one key enzyme of glycolysis process [36]. Allosteric activators including AMP, ADP and fructose-2, 6-bisphosphate (FRU-2, 6-P2) can bind to PFK-1 to increase the activity of PFK-1, among which FRU-2, 6-P2 is the most effective one [37, 38]. Meanwhile, the protein encoded by PFKFB3 can promote the synthesis of FRU-2, 6-P2 to increase its concentration in the microenvironment, which indirectly enhances glycolysis. In ALK (anaplastic lymphoma kinase)-positive non-small cell lung cancer, PFKFB3 is a downstream molecule of ALK-STAT3 signaling pathway that positively regulates the glycolysis level and plays a carcinogenic role in tumor cells [39]. The PFKFB3/AKT/ERCC1 (Excision repair cross-complementation group 1) pathway has been reported to promote the progression of hepatocellular carcinoma by enhancing DNA repair in the process of glycolysis [40]. The Kaplan Meier plotter is a powerful database to explore the correlation between the expression of particular gene and survival in more than 30,000 samples from 21 tumor types including breast cancer. The results of survival analysis presented in this study are based on Kaplan–Meier plotter database with strong reliability. Our results suggested that PFKFB3 was overexpressed and promoted the proliferation as well as migration in breast cancer with diabetes.

MicroRNA has become a novel research focus in recent years and miRNAs targeting PFKFB3 deserves further study. In this research, we found that miR-26 was the most probable upstream regulatory factor of PFKFB3 by comprehensive analysis of TargetScan and OncomiR online databases, which was further verified in MiRcode database. The tumor suppressive effect of miR-26/PFKFB3 has been confirmed in osteosarcoma, in which miR-26b inhibits the proliferation and metastasis of osteosarcoma cells and stimulates cell apoptosis by inducing PFKFB3 downregulation. The concentration of glycolysis-related molecules such as GLUT-1 also decreases correspondingly [41]. In addition, miR-26/PFKFB3 was also shown to play a similar role in gastric cancer patients with diabetes [42]. In our study, mediums with different concentration of glucose were utilized to confirm the induction of high glucose on PFKFB3 expression. Our results also indirectly indicated that high glucose upregulated PFKFB3 expression by miR-26 downregulation. To further explore the function of PFKFB3 in breast Cancer, GSEA was conducted to screen out several pathways with statistical significance in GSCA. Our results indicated that the promoting effect of PFKFB3 on epithelial-mesenchymal transformation in breast cancer is compelling and the RAS/MAPK is especially a statistically recognized possible pathway. MAPK is a well-known signaling pathway in cellular molecular biology that regulates cellular biological behaviors [43, 44]. Abnormally activated MAPK/ERK pathway have been found in a variety of tumors [45]. The MAPK/ERK pathway has been reported to negatively affect the prognosis of breast cancer and is associated with the adriamycin-resistance of breast cancer [46, 47]. As previously mentioned, metformin, a first-line drug for diabetes, was previously found to inhibit the development of breast cancer and improve the survival of breast cancer patients after immunotherapy [35]. Interestingly, while exploring the specific mechanism of metformin in decreasing blood glucose level and even cancer inhibition, some researchers found that MAPK signaling pathway could be inhibited by metformin and pancreatic aquaporin 7 (AQP7) was then reactivated to allow insulin secretion [48]. These results suggested that MAPK pathway may function in the regulation of glycolysis, which is corresponded with our results in this study.

The mechanism of poor prognosis in breast cancer patients with hyperglycemia is complex. Hyperglycemic environment has been demonstrated to trigger the HIF1 pathway by upregulating the expression of HIF1-ɑ, which ultimately leads to anti-apoptotic cell response. Excessive secretion of insulin can stimulate the synthesis of insulin-like growth factor (IGF-1), which can promote cell mitosis and inhibit apoptosis [49]. In addition, insulin resistance leads to an increase in free estrogen, which has been linked to postmenopausal breast cancer, by inhibiting the production of sex hormone-binding proteins [50]. In this study, we proved the cancer-promoting effect of PFKFB3 in hyperglycemic breast cancer cells by regulating PFKFB3 expression starting from glycolysis pathway, but there are still some limitations: first, it might be inappropriate to simulate the hyperglycemic environment in the human body with hyperglucose mediums; second, the regulatory effects of PFKFB3 on RAS/MAPK pathway should be confirmed by co-immunoprecipitation assay; last, an hyperglycemic animal model should be established to further verify the results in vitro.

## Conclusion

In conclusion, our study confirmed that PFKFB3 expression was correlated with the glucose level, lymph node metastasis, and tumor stage of breast cancer patients. Besides, hyperglycemia might enhance PFKFB3 expression by miR-26 down-regulation to promote the proliferation and migration of breast cancer via activating RAS/MAPK signaling pathway. Appropriate management of blood glucose level is vital for improving the prognosis of breast cancer patients with hyperglycemia.

## Data Availability

All data related to the results of this study are available within the article.

## Abbreviations

PFKFB3: 6-Phosphofructose-2-kinase/fructose-2, 6-bisphosphatase 3
EMT: Epithelial–mesenchymal transition
t/p-ERK: Total/phosphorylated-Extracellular regulated protein kinases
WAT: White adipose tissue
TNM: Tumor node metastasis
IHC: Immunohistochemistry
GEO: Gene Expression Omnibus
GSCA: Gene Set Cancer Analysis
WB: Western blot
NC: Negative control
BC: Breast cancer
PFS: Progression-free survival
OS: Overall survival
ALK: Anaplastic lymphoma kinase
ERCC1: Excision repair cross-complementation group 1
